# The Development of Large Radicular Cysts in Endodontically Versus Non-Endodontically Treated Maxillary Teeth

**DOI:** 10.3390/medicina57090991

**Published:** 2021-09-20

**Authors:** Ruth Schvartzman Cohen, Tomer Goldberger, Ina Merzlak, Igor Tsesis, Gavriel Chaushu, Gal Avishai, Eyal Rosen

**Affiliations:** 1 Oral and Maxillofacial Surgery Department—Rabin Medical Center, Beilinson Campus, 39 Ze’ev Jabotinsky St, Petach Tikvah 4941492, Israel; gavrielce@clalit.org.il (G.C.); drgalavishai@gmail.com (G.A.); 2 Department of Endodontics, School of Dental Medicine, Tel Aviv University, Ramat Aviv 6997801, Israel; dr.inamerzlak@gmail.com (I.M.); zasis@post.tau.ac.il (I.T.); dr.eyalrosen@gmail.com (E.R.); 3 Department of Oral and Maxillofacial Surgery, School of Dental Medicine, Tel Aviv University, Ramat Aviv 6997801, Israel

**Keywords:** maxillary, radicular cyst, endodontically treated teeth

## Abstract

*Background and Objectives*: Large radicular cysts of the maxilla present a clinical challenge, as they may cause recurrent infection, severe alveolar bone loss and disruption of the nasal and maxillary sinus floors. The aim of this study was to evaluate the effect of previous root canal treatment on the clinical presentation of large maxillary radicular cysts. *Materials and Methods*: All cases of radicular cysts treated at the Oral and Maxillofacial Surgery Department of a tertiary public hospital over a period of six years (2012–2018) were evaluated. Histologically confirmed radicular cysts of the maxilla with a maximal dimension of over 15 mm were included. Demographic data of the patients, clinical presentation and radiographic features of the lesions were analyzed. *Results*: A total of 211 inflammatory cysts were treated in the study period, of these 54 histologically diagnosed radicular cysts in the maxilla were found to have a maximal dimension of over 15 mm. The mean age of patients with large maxillary radicular cysts was 43.3 years, 57.6% of which were male and 42.4% female. The lateral incisor was the most common tooth affected (46.3%). The mean size of the large radicular cysts was 25 mm. Then, 83.8% of the cysts were observed in teeth with previous endodontic treatment. Teeth without endodontic treatment presented clinically with significantly fewer acute symptoms in comparison to teeth with previous endodontic treatment. *Conclusions*: the vast majority (83.8%) of large maxillary radicular cysts were associated with endodontically treated teeth. Previous endodontic treatment was correlated to increased frequency of clinical symptoms.

## 1. Introduction

Periapical lesions of the jaws are most commonly of endodontic origin and are related to pulp infection [1,2]. Most lesions of endodontic origin can be classified as either periapical granuloma or radicular cyst. The reported prevalence of radicular cysts within periapical lesions varies between 6–55% [2,3]. In an analysis of 2030 cystic lesions of the jaws, the prevalence of radicular cysts was 42% and the most common diagnosis of cysts of the jaws [4].

In the 2017 edition of the World Health Organization (WHO) classification of odontogenic lesions, radicular cysts were included in the inflammatory cyst group. A subclassification of this category includes radicular cyst (apical and periodontal cysts) and inflammatory collateral cysts (paradental cyst, buccal bifurcation cyst) [5].

Radicular cysts are a sequel of a chronically inflamed granulation tissue (the periapical granuloma) located adjacent to the apex of either endodontically or non-endodontically treated teeth with an infected root canal system [2,6,7]. However, the effects of a previous root canal treatment on the clinical presentation of these cysts, such as the size of the lesion size and the clinical symptoms, are still unclear.

It has been reported that radicular cysts are more frequently found in the maxilla [8], occurring almost ten times more frequently than in the mandible [9]. Most radicular cysts range in size from 5 to 15 mm. However, in the maxilla, a cyst may enlarge more than 15 mm [10,11,12,13]).

Thus, the development of these large maxillary cysts, and the effect of a previous root canal treatment on the clinical presentation is of a particular interest.

In this study, we retrospectively collected all cases of maxillary radicular cysts referred to a Oral and Maxillofacial Surgery Department of a large tertiary public hospital (Rabin Medical Center, Petach Tikva, Israel), over a period of 6 years, in order to evaluate the effect of a previous root canal treatment on the clinical presentation of large maxillary radicular cysts.

## 2. Materials and Methods

A retrospective review of all the radicular cysts treated at the Oral Maxillofacial Surgery Department of Rabin Medical Center, Petach Tikva, Israel for a period of 6 years (November 2012–November 2018). The study was approved by the institutional review board of authors’ hospital (00-88-19RMC).

The search was done based on pathological diagnostic code (Radicular Cyst) and according to the WHO classification of odontogenic lesions, subclassification radicular cyst, our search did not include other inflammatory collateral cysts such as paradental and buccal bifurcation cysts. Data was classified by the main investigator and the following characteristics were collected: age at diagnosis, gender, history of previous root canal treatment (yes/no), presence of swelling or expansion (yes/no), tooth location (divided to: central\lateral incisor; Canine; 1st\2nd premolar\molar teeth), largest dimension, presence of periapical lesions in other teeth, involvement of nasal floor and treatment performed (endodontic treatment/apicoectomy/enucleation/extraction/decompression/combination of treatments).

The cysts were measured by the main investigator on panoramic radiographs or cone bean computerized tomography (CBCT). The size of the periapical lesions was recorded at their widest horizontal or vertical diameters and the presence or absence of a radiopaque lamina of the periapical lesions was recorded.

Biopsy procedure was either incisional or excisional depending on the size of the lesion. The specimens were fixed in 4% neutral-buffered formaldehyde and processed for paraffin embedding. Specimens were sectioned and stained with hematoxylin and eosin using standard techniques. All the samples were analyzed by the same oral pathologist.

Radicular cysts were diagnosed histologically by demonstrating a lumen at least partially lined by stratified squamous epithelium and a connective tissue wall with various amounts of chronic and acute inflammatory cells [7].

Patients were divided in two groups. The first group included patients with a radicular cyst from a tooth with previous endodontic treatment (study group). The second group included patients with a radicular cyst without a previous endodontic treatment (comparison group).

Data analysis was perform using the Statistical Analysis System software (SAS^©^, Cary, NC, USA). T-test and Fisher’s exact test were used for grouped variables.

### 2.1. Inclusion Criteria

1. Over 18 years old.
2. Cyst of the maxilla with at least 15 mm diameter in panoramic radiography or CBCT.
3. Histological confirmation of a radicular cyst.

### 2.2. Exclusion Criteria

1. Odontogenic cysts that are not associated with a tooth with endodontic infection.
2. Cyst without clinicopathological correlation.
3. Residual cyst.

## 3. Results

### Demographic Data and Clinical Characteristics

A total of 211 inflammatory cysts were treated over a period of 6 years. Of these patients, 57 were diagnosed with inflammatory cysts of the maxilla (3 residual and 54 radicular cysts). According to the WHO classification of odontogenic lesions, residual and radicular cyst are included in the same subclassification, t. Residual cysts were excluded from this point because it was not clear which teeth caused the initial infection.

The patients age ranged between 19 years to 70 years, with a mean age of 43.3 years.

Regarding gender distribution of the 54 patients, 24 were male and 30 were female.

**Figure 1:**
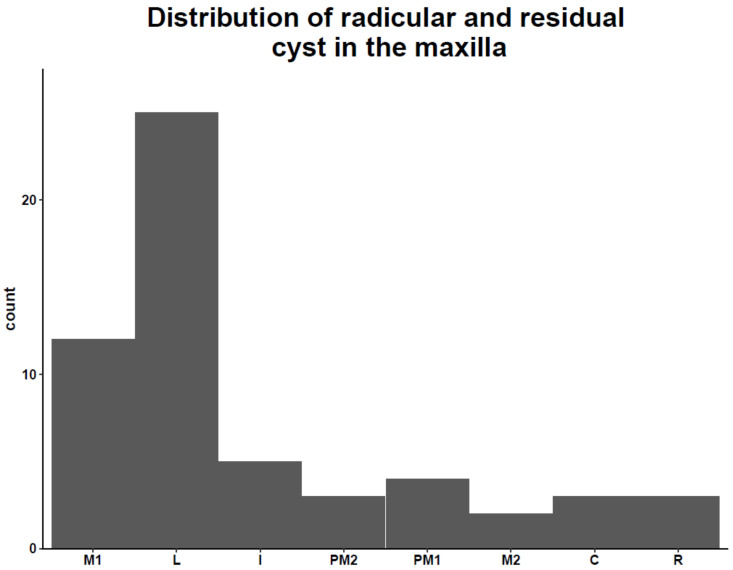
M1: First molar. L: lateral incisor. I: Central incisor. PM2: Second premolar. PM1 First premolar. M2: Second molar. C: canine. R: residual cyst.

The lateral incisor was the most common tooth affected in the maxilla in 25 of the 54 cases (46.3%), first molar 12 of 54 cases (22.2%) and central incisor and first premolar equally involved in 5 cases (9.2%), the distribution between the affected teeth is presented in Figure 1. In 20 cases, other periapical lesions were diagnosed in different teeth, and in 4 cases, bilateral lesions were found.

**Figure 2:**
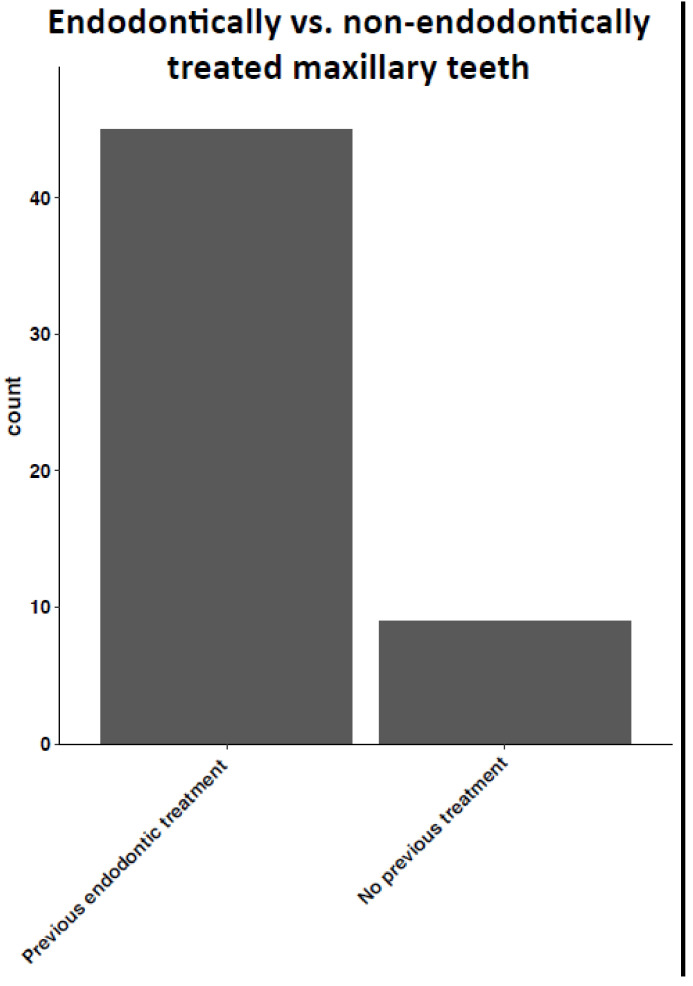
Radicular cyst found in endodontically versus non-endodontically treated maxillary teeth.

**Figure 3:**
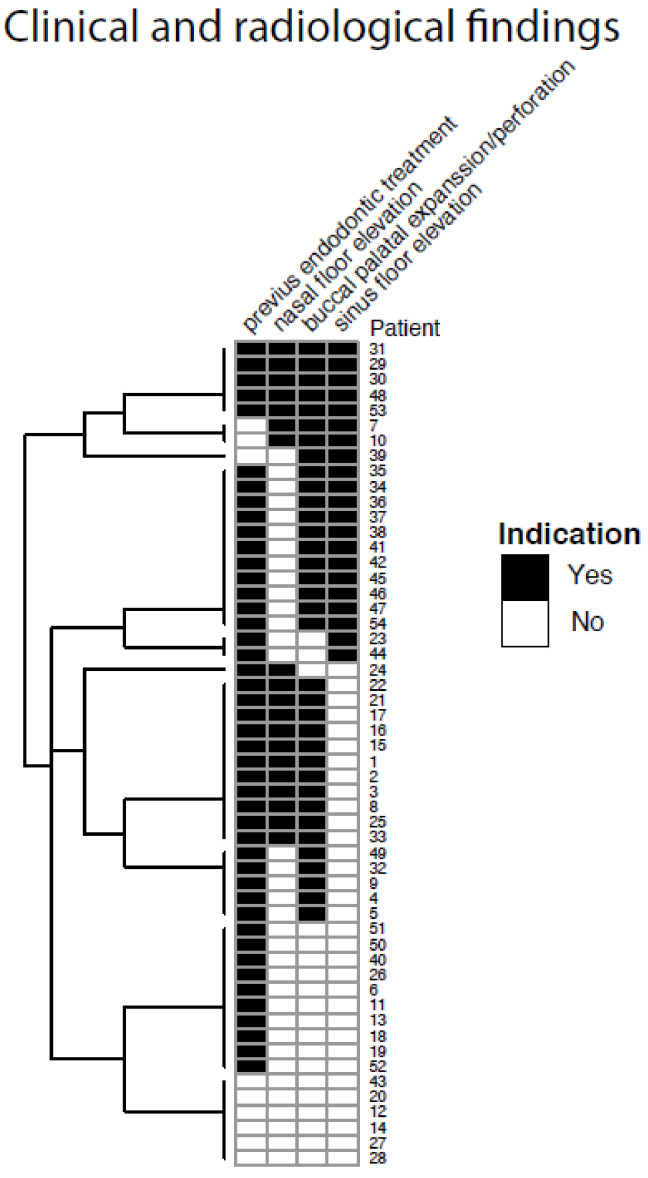
A cluster with representation of all patients clinical and radiological findings.

Having a history of root canal treatment was a major characteristic found in 45 of 54 patients (83.8%), Figure 2. This finding was statistically significant in both women and men of all ages, *p*-value for Char (Fisher) 0.286 Group 2, without endodontic treatment presented clinically with fewer acute symptoms at diagnosis in comparison to Group 1. A representation of all patients clinical and radiological findings can be seen in a Figure 3, and the most common finding was endodontically treated teeth.

## 4. Treatment

All the patients underwent biopsy for histological diagnosis. Treatment modalities included: endodontic treatment with decompression, extraction and decompression, apicoectomy and enucleation, extraction and enucleation, extraction enucleation and bone graft. The treatment selection was based on dental status, size of the cyst and nasal floor or sinus involvement. In 2 cases in which the size of the cyst was 35 mm and 25 mm, oro-nasal fistula presented as a minor complication that was treated surgically in a second stage.

## 5. Discussion

This retrospective study focused on the effect of previous root canal treatment on the clinical presentation of large maxillary radicular cysts that were diagnosed and surgically treated in over period of six years in one medical center. The radicular cyst is the most common cyst of the jaws and probably one of the most common lesions in the jaws. Tamiolakis et al. recently reported that 57% of 5294 jaw cysts diagnosed in a single Oral Pathology Department during a 38-year period were radicular cysts [6].

In most cases of radicular cysts of the maxilla, the involved tooth had been previously treated endodontically; however, from the results of the present study, it is impossible to determine the role of the root canal treatment on the etiology of the radicular cyst. Radicular cysts are formed in association with a tooth with infected root canal system [3,7,14,15,16,17,18,19,20,21,22,23,24,25]. These root canal infections may be divided into primary and secondary infections (Primary infections develop after an initial bacterial invasion into the pulp of non-endodontically treated teeth, while secondary infections occur following a previous root canal treatment (i.e., in endodontically treated teeth)) [26]. The bacterial flora found in endodontically treated teeth completely differs in its phenotype and characteristics and is much more resistant and able to survive harsh conditions compared to the flora found in non-endodontically treated teeth [27]. However, the effect of the type of root canal infection on the development of radicular cysts, and on their clinical presentation remain unclear.

The different composition of the endodontic flora and the interaction between different species is a subject of study in the literature and is believed to be responsible for the immunological host response and progression from apical granuloma to radicular cyst [8,9,10].

Mortensen et al., 1970 [12], indicated that lesions larger than 15–20 mm can be safely classified as cysts. However, various studies have indicated that basing diagnoses on radiographic analysis is not sufficient [28]. Matsuda et al. [29] concluded that it is not possible to confirm the diagnosis of lesions only through clinical and radiographic examination. In the current study, all cysts were confirmed histologically.

We have not included data about the frequency of all apical periodontitis lesions because most apical granulomas and sometimes radicular cyst are treated with conventional endodontic therapy without the need for biopsy [30], so this did not match our inclusion criteria.

The results of our study confirm the results of other studies in which the anterior part of the maxilla is more commonly affected [6]. No significant difference was found between females and males. Neither significant difference was found in age or size of the lesion between the two groups. The average age of diagnosis of radicular cyst was 43.4 years, this result is comparable to the results of Lo Muzio with a mean age of 38.4 [4]. The lateral incisor was the most frequent tooth overall.

## 6. Conclusions

In our study, 83.8% of all large maxillary radicular cysts were diagnosed from a tooth with previous endodontic treatment, this finding was not reported in the literature. Moreover, the group with radicular cysts with previous endodontic treatment was prone to chronic infection and swelling in more cases than the group without endodontic treatment. The common profile that was identified in the current study was of a large radicular cyst associated with a symptomatic anterior lateral root canal treated tooth. New studies on the possible effects of the microbiology of endodontically and non-endodontically treated teeth are needed.
